# Supplementary material for: Pyruvate Dehydrogenase Kinase 1 inhibition mediated oxidative phosphorylation enhancement in cartilage promotes osteoarthritis progression
Source: BMC Musculoskelet Disord. 2023 Jul 20;24:597. doi: 10.1186/s12891-023-06585-6 (PMC10357736; doi:10.1186/s12891-023-06585-6)
Supplement: Supplementary file 1 — Additional file 1. [file 12891_2023_6585_MOESM1_ESM.zip › KEGG.Gsea.1653622432464/KEGG_TGF_BETA_SIGNALING_PATHWAY.html]

Details for gene set KEGG\_TGF\_BETA\_SIGNALING\_PATHWAY[GSEA]

|  || Dataset | 55457\_collapsed\_to\_symbols.cls.cls #OA\_versus\_normal.cls.cls #OA\_versus\_normal\_repos |
| Phenotype | cls.cls#OA\_versus\_normal\_repos |
| Upregulated in class | normal |
| GeneSet | KEGG\_TGF\_BETA\_SIGNALING\_PATHWAY |
| Enrichment Score (ES) | -0.32521904 |
| Normalized Enrichment Score (NES) | -1.3537824 |
| Nominal p-value | 0.051805336 |
| FDR q-value | 0.2591677 |
| FWER p-Value | 0.999 |
Table: GSEA Results Summary

  

Fig 1: Enrichment plot: KEGG\_TGF\_BETA\_SIGNALING\_PATHWAY      
 Profile of the Running ES Score & Positions of GeneSet Members on the Rank Ordered List

  

| SYMBOL | TITLE | RANK IN GENE LIST | RANK METRIC SCORE | RUNNING ES | CORE ENRICHMENT || 1 | SMAD2 | SMAD family member 2 [Source:HGNC Symbol;Acc:HGNC:6768] | 275 | 0.768 | 0.0094 | No |
| 2 | RBX1 | ring-box 1 [Source:HGNC Symbol;Acc:HGNC:9928] | 348 | 0.724 | 0.0329 | No |
| 3 | COMP | cartilage oligomeric matrix protein [Source:HGNC Symbol;Acc:HGNC:2227] | 415 | 0.700 | 0.0559 | No |
| 4 | MAPK3 | mitogen-activated protein kinase 3 [Source:HGNC Symbol;Acc:HGNC:6877] | 757 | 0.579 | 0.0526 | No |
| 5 | GDF7 | growth differentiation factor 7 [Source:HGNC Symbol;Acc:HGNC:4222] | 928 | 0.533 | 0.0607 | No |
| 6 | TFDP1 | transcription factor Dp-1 [Source:HGNC Symbol;Acc:HGNC:11749] | 1048 | 0.508 | 0.0718 | No |
| 7 | LTBP1 | latent transforming growth factor beta binding protein 1 [Source:HGNC Symbol;Acc:HGNC:6714] | 1899 | 0.373 | 0.0205 | No |
| 8 | TNF | tumor necrosis factor [Source:HGNC Symbol;Acc:HGNC:11892] | 2098 | 0.350 | 0.0191 | No |
| 9 | ID3 | "inhibitor of DNA binding 3, HLH protein [Source:HGNC Symbol;Acc:HGNC:5362]" | 2309 | 0.325 | 0.0158 | No |
| 10 | THBS4 | thrombospondin 4 [Source:HGNC Symbol;Acc:HGNC:11788] | 2349 | 0.320 | 0.0257 | No |
| 11 | LEFTY2 | left-right determination factor 2 [Source:HGNC Symbol;Acc:HGNC:3122] | 2406 | 0.314 | 0.0339 | No |
| 12 | THBS3 | thrombospondin 3 [Source:HGNC Symbol;Acc:HGNC:11787] | 2474 | 0.305 | 0.0409 | No |
| 13 | TGFB1 | transforming growth factor beta 1 [Source:HGNC Symbol;Acc:HGNC:11766] | 2490 | 0.303 | 0.0520 | No |
| 14 | BMPR1A | bone morphogenetic protein receptor type 1A [Source:HGNC Symbol;Acc:HGNC:1076] | 2745 | 0.275 | 0.0432 | No |
| 15 | ID1 | "inhibitor of DNA binding 1, HLH protein [Source:HGNC Symbol;Acc:HGNC:5360]" | 3349 | 0.216 | 0.0049 | No |
| 16 | SMAD6 | SMAD family member 6 [Source:HGNC Symbol;Acc:HGNC:6772] | 3460 | 0.206 | 0.0046 | No |
| 17 | ACVR1 | activin A receptor type 1 [Source:HGNC Symbol;Acc:HGNC:171] | 3898 | 0.165 | -0.0229 | No |
| 18 | SMAD3 | SMAD family member 3 [Source:HGNC Symbol;Acc:HGNC:6769] | 4061 | 0.150 | -0.0295 | No |
| 19 | PPP2CA | protein phosphatase 2 catalytic subunit alpha [Source:HGNC Symbol;Acc:HGNC:9299] | 4146 | 0.143 | -0.0303 | No |
| 20 | E2F5 | E2F transcription factor 5 [Source:HGNC Symbol;Acc:HGNC:3119] | 4542 | 0.110 | -0.0567 | No |
| 21 | INHBC | inhibin subunit beta C [Source:HGNC Symbol;Acc:HGNC:6068] | 4788 | 0.088 | -0.0723 | No |
| 22 | THBS2 | thrombospondin 2 [Source:HGNC Symbol;Acc:HGNC:11786] | 4943 | 0.077 | -0.0812 | No |
| 23 | AMH | anti-Mullerian hormone [Source:HGNC Symbol;Acc:HGNC:464] | 5042 | 0.070 | -0.0861 | No |
| 24 | TGFBR1 | transforming growth factor beta receptor 1 [Source:HGNC Symbol;Acc:HGNC:11772] | 5280 | 0.050 | -0.1026 | No |
| 25 | RPS6KB2 | ribosomal protein S6 kinase B2 [Source:HGNC Symbol;Acc:HGNC:10437] | 5496 | 0.033 | -0.1180 | No |
| 26 | LEFTY1 | left-right determination factor 1 [Source:HGNC Symbol;Acc:HGNC:6552] | 5935 | -0.000 | -0.1522 | No |
| 27 | BMPR1B | bone morphogenetic protein receptor type 1B [Source:HGNC Symbol;Acc:HGNC:1077] | 5959 | -0.002 | -0.1539 | No |
| 28 | ACVR2A | activin A receptor type 2A [Source:HGNC Symbol;Acc:HGNC:173] | 5960 | -0.002 | -0.1538 | No |
| 29 | GDF5 | growth differentiation factor 5 [Source:HGNC Symbol;Acc:HGNC:4220] | 6205 | -0.021 | -0.1720 | No |
| 30 | TGFB3 | transforming growth factor beta 3 [Source:HGNC Symbol;Acc:HGNC:11769] | 6293 | -0.027 | -0.1777 | No |
| 31 | IFNG | interferon gamma [Source:HGNC Symbol;Acc:HGNC:5438] | 6410 | -0.036 | -0.1853 | No |
| 32 | SMAD5 | SMAD family member 5 [Source:HGNC Symbol;Acc:HGNC:6771] | 6530 | -0.045 | -0.1928 | No |
| 33 | BMP8A | bone morphogenetic protein 8a [Source:HGNC Symbol;Acc:HGNC:21650] | 6537 | -0.045 | -0.1915 | No |
| 34 | RBL2 | RB transcriptional corepressor like 2 [Source:HGNC Symbol;Acc:HGNC:9894] | 6764 | -0.060 | -0.2067 | No |
| 35 | MAPK1 | mitogen-activated protein kinase 1 [Source:HGNC Symbol;Acc:HGNC:6871] | 6853 | -0.068 | -0.2108 | No |
| 36 | TGFB2 | transforming growth factor beta 2 [Source:HGNC Symbol;Acc:HGNC:11768] | 6985 | -0.079 | -0.2179 | No |
| 37 | ZFYVE9 | zinc finger FYVE-type containing 9 [Source:HGNC Symbol;Acc:HGNC:6775] | 7049 | -0.083 | -0.2194 | No |
| 38 | SMAD7 | SMAD family member 7 [Source:HGNC Symbol;Acc:HGNC:6773] | 7231 | -0.098 | -0.2296 | No |
| 39 | PPP2CB | protein phosphatase 2 catalytic subunit beta [Source:HGNC Symbol;Acc:HGNC:9300] | 7265 | -0.100 | -0.2282 | No |
| 40 | INHBE | inhibin subunit beta E [Source:HGNC Symbol;Acc:HGNC:24029] | 7693 | -0.134 | -0.2561 | No |
| 41 | RBL1 | RB transcriptional corepressor like 1 [Source:HGNC Symbol;Acc:HGNC:9893] | 7776 | -0.140 | -0.2569 | No |
| 42 | BMP4 | bone morphogenetic protein 4 [Source:HGNC Symbol;Acc:HGNC:1071] | 7816 | -0.142 | -0.2542 | No |
| 43 | PITX2 | paired like homeodomain 2 [Source:HGNC Symbol;Acc:HGNC:9005] | 8137 | -0.166 | -0.2725 | No |
| 44 | ID4 | "inhibitor of DNA binding 4, HLH protein [Source:HGNC Symbol;Acc:HGNC:5363]" | 8174 | -0.169 | -0.2685 | No |
| 45 | TGFBR2 | transforming growth factor beta receptor 2 [Source:HGNC Symbol;Acc:HGNC:11773] | 8241 | -0.174 | -0.2667 | No |
| 46 | E2F4 | E2F transcription factor 4 [Source:HGNC Symbol;Acc:HGNC:3118] | 8445 | -0.190 | -0.2749 | No |
| 47 | SKP1 | S-phase kinase associated protein 1 [Source:HGNC Symbol;Acc:HGNC:10899] | 8454 | -0.190 | -0.2679 | No |
| 48 | BMP5 | bone morphogenetic protein 5 [Source:HGNC Symbol;Acc:HGNC:1072] | 8488 | -0.193 | -0.2627 | No |
| 49 | FST | follistatin [Source:HGNC Symbol;Acc:HGNC:3971] | 8511 | -0.194 | -0.2566 | No |
| 50 | RHOA | ras homolog family member A [Source:HGNC Symbol;Acc:HGNC:667] | 8601 | -0.201 | -0.2555 | No |
| 51 | SMAD4 | SMAD family member 4 [Source:HGNC Symbol;Acc:HGNC:6770] | 9035 | -0.235 | -0.2798 | No |
| 52 | BMP7 | bone morphogenetic protein 7 [Source:HGNC Symbol;Acc:HGNC:1074] | 9544 | -0.278 | -0.3083 | No |
| 53 | CUL1 | cullin 1 [Source:HGNC Symbol;Acc:HGNC:2551] | 9762 | -0.297 | -0.3133 | Yes |
| 54 | SMAD1 | SMAD family member 1 [Source:HGNC Symbol;Acc:HGNC:6767] | 9905 | -0.312 | -0.3118 | Yes |
| 55 | BMP6 | bone morphogenetic protein 6 [Source:HGNC Symbol;Acc:HGNC:1073] | 10021 | -0.323 | -0.3078 | Yes |
| 56 | RPS6KB1 | ribosomal protein S6 kinase B1 [Source:HGNC Symbol;Acc:HGNC:10436] | 10224 | -0.343 | -0.3097 | Yes |
| 57 | CDKN2B | cyclin dependent kinase inhibitor 2B [Source:HGNC Symbol;Acc:HGNC:1788] | 10324 | -0.353 | -0.3033 | Yes |
| 58 | CHRD | chordin [Source:HGNC Symbol;Acc:HGNC:1949] | 10355 | -0.356 | -0.2913 | Yes |
| 59 | PPP2R1A | protein phosphatase 2 scaffold subunit Aalpha [Source:HGNC Symbol;Acc:HGNC:9302] | 10395 | -0.360 | -0.2798 | Yes |
| 60 | BMP8B | bone morphogenetic protein 8b [Source:HGNC Symbol;Acc:HGNC:1075] | 10419 | -0.363 | -0.2670 | Yes |
| 61 | AMHR2 | anti-Mullerian hormone receptor type 2 [Source:HGNC Symbol;Acc:HGNC:465] | 10571 | -0.381 | -0.2635 | Yes |
| 62 | PPP2R1B | protein phosphatase 2 scaffold subunit Abeta [Source:HGNC Symbol;Acc:HGNC:9303] | 10578 | -0.382 | -0.2487 | Yes |
| 63 | ZFYVE16 | zinc finger FYVE-type containing 16 [Source:HGNC Symbol;Acc:HGNC:20756] | 10585 | -0.382 | -0.2338 | Yes |
| 64 | ROCK2 | Rho associated coiled-coil containing protein kinase 2 [Source:HGNC Symbol;Acc:HGNC:10252] | 10648 | -0.388 | -0.2230 | Yes |
| 65 | CREBBP | CREB binding protein [Source:HGNC Symbol;Acc:HGNC:2348] | 10881 | -0.414 | -0.2245 | Yes |
| 66 | BMP2 | bone morphogenetic protein 2 [Source:HGNC Symbol;Acc:HGNC:1069] | 10919 | -0.420 | -0.2105 | Yes |
| 67 | ACVR2B | activin A receptor type 2B [Source:HGNC Symbol;Acc:HGNC:174] | 11011 | -0.431 | -0.2003 | Yes |
| 68 | BMPR2 | bone morphogenetic protein receptor type 2 [Source:HGNC Symbol;Acc:HGNC:1078] | 11174 | -0.454 | -0.1947 | Yes |
| 69 | INHBA | inhibin subunit beta A [Source:HGNC Symbol;Acc:HGNC:6066] | 11237 | -0.462 | -0.1809 | Yes |
| 70 | DCN | decorin [Source:HGNC Symbol;Acc:HGNC:2705] | 11402 | -0.487 | -0.1742 | Yes |
| 71 | THBS1 | thrombospondin 1 [Source:HGNC Symbol;Acc:HGNC:11785] | 11498 | -0.501 | -0.1614 | Yes |
| 72 | SMAD9 | SMAD family member 9 [Source:HGNC Symbol;Acc:HGNC:6774] | 11633 | -0.522 | -0.1509 | Yes |
| 73 | EP300 | E1A binding protein p300 [Source:HGNC Symbol;Acc:HGNC:3373] | 11741 | -0.540 | -0.1376 | Yes |
| 74 | SMURF2 | SMAD specific E3 ubiquitin protein ligase 2 [Source:HGNC Symbol;Acc:HGNC:16809] | 11802 | -0.550 | -0.1202 | Yes |
| 75 | ID2 | inhibitor of DNA binding 2 [Source:HGNC Symbol;Acc:HGNC:5361] | 11852 | -0.560 | -0.1015 | Yes |
| 76 | ROCK1 | Rho associated coiled-coil containing protein kinase 1 [Source:HGNC Symbol;Acc:HGNC:10251] | 12046 | -0.605 | -0.0922 | Yes |
| 77 | ACVRL1 | activin A receptor like type 1 [Source:HGNC Symbol;Acc:HGNC:175] | 12242 | -0.656 | -0.0810 | Yes |
| 78 | SMURF1 | SMAD specific E3 ubiquitin protein ligase 1 [Source:HGNC Symbol;Acc:HGNC:16807] | 12344 | -0.688 | -0.0613 | Yes |
| 79 | SP1 | Sp1 transcription factor [Source:HGNC Symbol;Acc:HGNC:11205] | 12557 | -0.777 | -0.0466 | Yes |
| 80 | INHBB | inhibin subunit beta B [Source:HGNC Symbol;Acc:HGNC:6067] | 12680 | -0.851 | -0.0219 | Yes |
| 81 | MYC | "MYC proto-oncogene, bHLH transcription factor [Source:HGNC Symbol;Acc:HGNC:7553]" | 12801 | -0.970 | 0.0077 | Yes |
Table: GSEA details [plain text format]

  

Fig 2: KEGG\_TGF\_BETA\_SIGNALING\_PATHWAY      
 Blue-Pink O' Gram in the Space of the Analyzed GeneSet

  

Fig 3: KEGG\_TGF\_BETA\_SIGNALING\_PATHWAY: Random ES distribution      
 Gene set null distribution of ES for **KEGG\_TGF\_BETA\_SIGNALING\_PATHWAY**
